# Serum Autotaxin Is a Parameter for the Severity of Liver Cirrhosis and Overall Survival in Patients with Liver Cirrhosis – A Prospective Cohort Study

**DOI:** 10.1371/journal.pone.0103532

**Published:** 2014-07-25

**Authors:** Thomas Pleli, Daniel Martin, Bernd Kronenberger, Friederike Brunner, Verena Köberle, Georgios Grammatikos, Harald Farnik, Yolanda Martinez, Fabian Finkelmeier, Sandra Labocha, Nerea Ferreirós, Stefan Zeuzem, Albrecht Piiper, Oliver Waidmann

**Affiliations:** 1 Medizinische Klinik 1, Schwerpunkt Gastroenterologie und Hepatologie, Universitätsklinikum Frankfurt, Goethe-Universität, Frankfurt, Germany; 2 pharmazentrum frankfurt/ZAFES, Institut für Klinische Pharmakologie, Universitätsklinikum Frankfurt, Frankfurt, Germany; University of Colorado, United States of America

## Abstract

**Background:**

Autotaxin (ATX) and its product lysophosphatidic acid (LPA) are considered to be involved in the development of liver fibrosis and elevated levels of serum ATX have been found in patients with hepatitis C virus associated liver fibrosis. However, the clinical role of systemic ATX in the stages of liver cirrhosis was unknown. Here we investigated the relation of ATX serum levels and severity of cirrhosis as well as prognosis of cirrhotic patients.

**Methods:**

Patients with liver cirrhosis were prospectively enrolled and followed until death, liver transplantation or last contact. Blood samples drawn at the day of inclusion in the study were assessed for ATX content by an enzyme-linked immunosorbent assay. ATX levels were correlated with the stage as well as complications of cirrhosis. The prognostic value of ATX was investigated by uni- and multivariate Cox regression analyses. LPA concentration was determined by liquid chromatography-tandem mass spectrometry.

**Results:**

270 patients were enrolled. Subjects with liver cirrhosis showed elevated serum levels of ATX as compared to healthy subjects (0.814±0.42 mg/l vs. 0.258±0.40 mg/l, P<0.001). Serum ATX levels correlated with the Child-Pugh stage and the MELD (model of end stage liver disease) score and LPA levels (r = 0.493, P = 0.027). Patients with hepatic encephalopathy (P = 0.006), esophageal varices (P = 0.002) and portal hypertensive gastropathy (P = 0.008) had higher ATX levels than patients without these complications. Low ATX levels were a parameter independently associated with longer overall survival (hazard ratio 0.575, 95% confidence interval 0.365–0.905, P = 0.017).

**Conclusion:**

Serum ATX is an indicator for the severity of liver disease and the prognosis of cirrhotic patients.

## Introduction

Liver cirrhosis is the result of chronic injury and fibrotic remodeling of the liver [1]. The most common causes of liver cirrhosis are alcohol abuse, chronic infections with hepatotropic viruses, namely hepatitis B (HBV) and hepatitis C (HCV) viruses and non-alcoholic steatohepatitis. Patients suffering from liver cirrhosis are at risk of decompensation which is associated with impaired prognosis. Cirrhosis specific complications that may arise include ascites, spontaneous bacterial peritonitis (SBP), hepatorenal syndrome (HRS), gastrointestinal bleeding, hepatic encephalopathy (HE) and hepatocellular carcinoma (HCC) [2].

Hepatic stellate cells (HSCs) are a major cell type involved in development of liver fibrosis [1]. In continuously injured livers hepatic stellate cells are activated and transdifferentiate into myofibroblasts, resulting in the production of abundant extracellular matrices [3]–[5]. Lysophosphatidic acid (LPA), which is formed from lysophosphatidylcholine by autotaxin (ATX), a secreted glycoprotein possessing both phosphodiesterase and lysophospholipase D activity [6], [7], activates hepatic stellate cells, stimulates their contraction and inhibits their apoptosis [8], [9], [10]. ATX is widely expressed in tissues such as brain, placenta or high endothelial venules [11]–[14]. In mice with heterozygous loss of the ATX gene the LPA plasma concentration was half of that in wild-type mice, whereas complete knock-out of ATX is embryonic lethal due to blood vessel abnormalities [15], [16]. Recently, a connection between liver fibrosis and serum or plasma LPA and ATX emerged in patients with chronic HCV infection [17], [18]. ATX activity or protein levels are also elevated in patients with malignant diseases including pancreatic cancer, follicular lymphoma and HCC [19]–[23]. However, to our knowledge serum ATX in different stages of liver cirrhosis and its prognostic value has not yet been investigated. Therefore, we here investigated if serum ATX might be an indicator for the severity of liver cirrhosis. Furthermore, we assessed if serum ATX levels might be associated with specific complications of liver cirrhosis as well as overall survival (OS). To this end we performed a prospective cohort study in patients with liver cirrhosis, assessed the serum ATX levels and correlated them with the prevalence of complications and OS.

## Methods

### Patients

Between May 2009 and June 2011 patients with liver cirrhosis, who were treated in our department were enrolled in the present study after giving written informed consent. Liver cirrhosis was assessed by means of histopathological examination of liver tissue or characteristic findings in ultrasound, computed tomography (CT) or magnetic resonance imaging (MRI). Exclusion criteria were the following: an age below 18 years, a history of solid organ transplantation and a history of malignant tumors other than HCC in the last five years.

Patients were assessed for symptoms of hepatic decompensation (esophageal varices, ascites, SBP, HRS and HE) at the day of inclusion. In order to evaluate the stage of liver cirrhosis Child-Pugh and MELD score were assessed using routine laboratory parameters and clinical examination. Examination of the upper gastrointestinal tract by esophagogastroduodenoscopy was recommended to all patients to assess the severity of portal hypertension. The study was approved by the Ethics Committee of Frankfurt University Hospital.

### Control cohort

85 healthy subjects (35 males and 50 females) without severe chronic disease were included in the control cohort and serum ATX levels were determined.

### Blood sampling and clinical chemistry

At the day of study inclusion blood samples were obtained from each subject. In order to remove remaining cells serum tubes were centrifuged at 1500 g for 10 min at 4°C, followed by a second centrifugation (2000 g for 10 min at 4°C). Thereafter, the serum samples were aliquoted and stored at −80°C. Routine laboratory parameters were measured at the Central Laboratory of the Frankfurt University Hospital.

### Detection of serum ATX levels by an enzyme-linked immunosorbent assay

Serum ATX levels were determined with the Quantikine human ATX immunoassay (R&D Systems, Minneapolis, Minnesota), according to the recommendation of the manufacturer. All measurements were performed on a Tecan SLT Rainbow Plate Reader (Tecan, Männedorf, Switzerland).

### Determination of LPA concentration by liquid chromatography-tandem mass spectrometry

LPA levels in serum were determined using liquid-liquid extraction prior analysis as described elsewhere [24], [25] and consisted of the sum of the LPA species 16∶0, 18∶0, 18∶1, 18∶2, 18∶3, 20∶4. Sample extraction was performed by liquid-liquid extraction. Fifty µl serum were spiked with the internal standard (10 µl LPA 17∶0, 500 ng/ml in methanol) and extracted twice with 500 µl of water saturated n-butanol. The combined organic phases were removed at a temperature of 45°C under a gentle stream of nitrogen. The residues were reconstituted with 50 µl of methanol, centrifuged for 1 min at 22,238 g, and the supernatants transferred to glass vials before injection (20 µl) into the liquid chromatography-tandem mass spectrometry (LC-MS/MS) system.

The LC-MS/MS system consisted of an hybrid triple quadrupole-ion trap mass spectrometer QTrap 5500 (AB Sciex, Darmstadt, Germany), an Agilent 1260 HPLC binary pump, column oven and degasser (Agilent, Waldbronn, Germany), and a HTC Pal autosampler (CTC Analytics, Zwingen, Switzerland). High-purity nitrogen for the mass spectrometer was produced by a NGM 22-LC-MS nitrogen generator (cmc Instruments, Eschborn, Germany).

The mass spectrometer was operated in the negative ion mode with an electrospray voltage of −4500 V at 350°C. Multiple reaction monitoring (MRM) was used for identification and quantification. The mass transitions used for quantitation were *m/z* 409.2→153.0 (declustering potential, DP, −80 V, collision energy, CE, −65 V, LPA 16∶0), *m/z* 423.2→269.3 (DP −110 V, CE −40 V, LPA 17∶0, internal standard), *m/z* 437.2→153.0 (DP −170 V, CE −45 V, LPA 18∶0), *m/z* 435.2→153.0 (DP −160 V, CE −70 V, LPA 18∶1), *m/z* 433.2→153.0 (DP −90 V, CE −45 V, LPA 18∶2), *m/z* 431.2→153.0 (DP −150 V, CE −65 V, LPA 18∶3) and *m/z* 457.2→153.0 (DP −165 V, CE −45 V, LPA 20∶4) all with a dwell time of 50 ms.

For the chromatographic separation, a Luna 5u C18 (2) Mercury column was used (20×2 mm inner diameter, 5 µm particle size, and 100 Å pore size (Phenomenex, Aschaffenburg, Germany) with the same material pre-column. A linear gradient was used at a flow rate of 0.4 ml/min for the separation of the analytes with a total run time of 7 min. Mobile phase A was 50 mM ammonium acetate containing 0.2% formic acid and mobile phase B was acetonitrile/isopropyl alcohol/formic acid (50∶50∶0.2, v/v/v). The gradient started with 60% A and was maintained for 0.5 min reaching 5% within another 0.5 min. These conditions were held for 2.5 min. The mobile phase shifted back to 60% A within 0.5 min, and was held for 3 min to re-equilibrate the column.

Quantification was performed with Analyst software version 1.5 (AB Sciex, Darmstadt, Germany) using the internal standard method. Ratios of analyte peak area and internal standard area (*y*-axis) were plotted against concentration (*x*-axis), and calibration curves (0.1 to 500 ng/ml each analyte) were calculated by linear regression with 1/x concentration weighting. The coefficient of correlation was at least 0.99. Variations in accuracy were less than 15% over the range of calibration.

### Statistical analysis

Data were analyzed using the BiAS software for Windows (version 10.04, Epsilon-Verlag, Darmstadt, Germany) and SPSS version 21 (IBM, Chicago, IL). In order to assess differences in serum biomarker values between different patient groups the non-parametric Wilcoxon-Mann-Whitney and Kruskal-Wallis tests were used. For comparing multiple subgroups a Bonferroni correction was used. P values<0.05 were considered to be significant. Relations between two variables were assessed using the Spearman rank correlation. In the box plots the vertical lines indicate the ranges and the horizontal boundaries of the boxes represent the first and third quartile. Diagnostic accuracies for cirrhosis were estimated using the receiver operating curve (ROC) analysis.

Survival rates were assessed using the Cox regression model. Survival curves were calculated with Cox regression models. Independent predictors of survival were determined with a multivariate Cox regression analysis using forward stepwise (likelihood ratio) entry. Death was considered as event. Follow-up time was time until death or last contact to the patient. In patients who underwent liver transplantation the date of liver transplantation was recorded as last contact. Liver transplantation was not considered as event.

## Results

270 patients with liver cirrhosis were prospectively included in this study. The patient characteristics are summarized in Table 1. Alcohol abuse and chronic HCV infection were the predominant causes for liver cirrhosis in this cohort. 199 patients showed signs of hepatic decompensation (ascites, HRS, HE, SBP or gastrointestinal bleeding) at the time of inclusion in the study. Moreover, 47 (17.3%) patients suffered from HCC. The mean follow-up time was 381±411 days. 38 patients were allocated to liver transplantation within the study time and were excluded from further analysis from the day of transplantation. 85 patients died within the study.

**Table 1:** Patients' characteristics.

Parameter	Male	female
**Epidemiology**		
Patients, n (%)	181 (67.0)	89 (33.0)
Age, median, range	58 (27–79)	55 (25–84)
**Etiology of liver disease**		
Alcohol abuse, n (%)	95 (52.5)	41 (46.1)
Hepatitis C, n (%)	45 (24.9)	28 (31.5)
Hepatitis B, n (%)	24 (13.3)	11 (12.4)
non-alcoholic steatohepatitis, n (%)	6 (3.3)	1 (1.1)
Hereditary hemochromatosis, n (%)	4 (2.2)	3 (3.4)
Cryptogenic, n (%)	16 (8.8)	8 (9.0)
Primary sclerosing cholangitis, n (%)	8 (4.4)	7 (7.9)
Primary biliary cirrhosis, n (%)	1 (0.6)	2 (2.2)
Autoimmune hepatitis, n (%)	3 (1.7)	6 (6.7)
**Child-Pugh stage**		
A, n (%)	42 (23.2)	14 (15.7)
B, n (%)	89 (49.2)	40 (44.9)
C, n (%)	50 (27.6)	35 (39.3)
**MELD** 1 **, median, range**	14 (6–40)	16 (7–36)
**Laboratory results**		
Leukocytes, median, range (µl^−1^)	5.25 (0.6–56.5)	5.16 (1.68–20.16)
Hemoglobin, median, range (g/dl)	10.9, 7–18	10.1, 6–15
Thrombocytes, median, range (nl^−1^)	97, 15–1507	100, 18–410
Sodium, median, range (mmol/l)	138, 111–148	139, 123–150
Creatinine, median, range (mg/dl)	1.03, 0.42–6.77	0.97, 0.38–5.00
Albumin median, range (mg/dl)	3.2, 1.7–5.2	3.2, 1.6–5.2
INR^2^, median, range	1.35, 0.89–3.07	1.47, 0.85–4.2
Bilirubin, median, range (mg/dl)	1.9, 0.2–26.8	2.2, 0.3–51.0
ALT^3^, median, range (U/l)	32, 2–1268	32, 7–1594
AST^4^, median, range (U/l)	53, 15–2823	52, 15–1176
GGT^5^, median, range (U/l)	112, 14–1178	82, 17–825
ALP^6^, median, range (U/l)	121, 34–422	116, 31–688

Abbreviations:

1 MELD, model of end stage liver disease;

2 INR, internationalized ratio;

3 ALT, alanine aminotransferase,

4 AST, aspartate aminotransferase;

5 GGT, gamma-glutaryl-transferase;

6 ALP, alkaline phosphatase.

### Patients with liver cirrhosis show elevated levels of serum ATX

**Figure 1:**
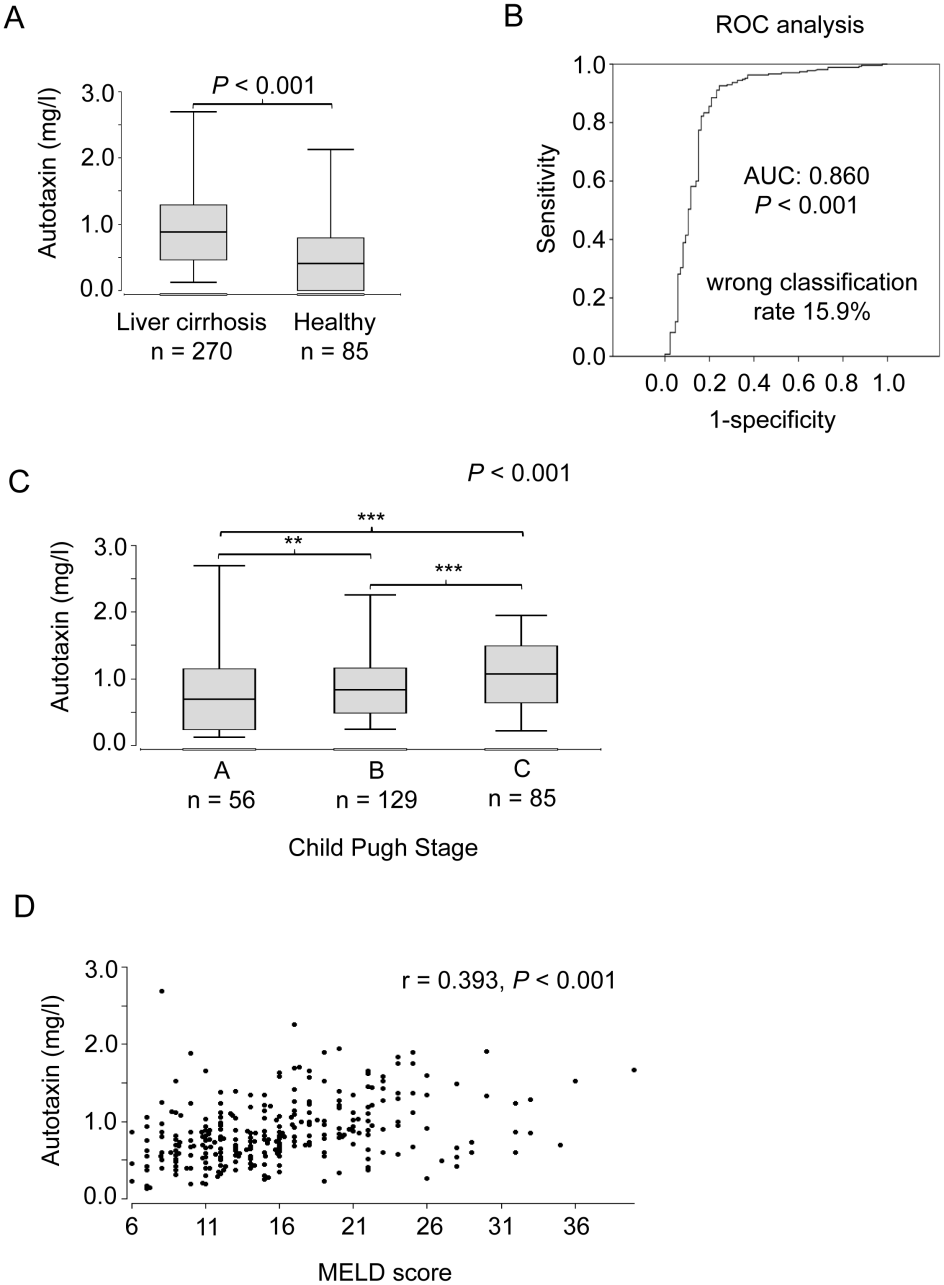
Serum autotaxin levels are elevated in liver cirrhosis and correlate with the stage of cirrhosis. (A) ATX serum concentrations in patients and in healthy subjects. P values referring to differences between the groups were calculated with the Wilcoxon-Mann-Whitney test. (B) AUROC curve for prediction of ATX in liver cirrhosis patients. (C) Serum ATX levels in patients with different Child-Pugh stages, P values referring to differences between the groups are calculated with the Kruskal-Wallis test (**P<0.01; ***P<0.001). The Bonferroni correction was used for the subgroup comparisons. (D) Correlation between MELD score and ATX serum levels in patients with liver cirrhosis. The correlation coefficient was calculated by the Spearman test.

Serum ATX levels were assessed in the 270 patients with liver cirrhosis and 85 healthy subjects. Patients with liver cirrhosis had significantly higher serum levels of ATX in comparison to the subjects of the control cohort (0.814±0.42 mg/l vs. 0.258±0.40 mg/l, P<0.001) (Figure 1A). As it has been reported that ATX levels show gender differences [26], ATX levels were compared between male and female cirrhotic patients. Indeed female patients had significantly higher serum ATX levels than male patients (0.858±0.43 mg/l vs. 0.770±0.41 mg/l, P = 0.011). A significant gender difference was also found in the healthy subjects (0.346±0.47 mg/l vs. 0.179±0.04 mg/l, females vs. males, respectively, P<0.001).

In order to investigate the ability of ATX to discriminate between healthy subjects and cirrhotic patients, a receiver operating characteristic (ROC) analysis was performed. The area under the ROC curves (AUROCs) was 0.860. The optimal cut-off value was 0.370 mg/l with a false classification rate of 15.9% (Figure 1B).

### Elevated serum ATX levels are associated with the severity of liver cirrhosis

It has been shown that serum ATX levels correlate with the stage of liver fibrosis in patients with chronic HCV infection [8]. To investigate if there is an association between serum ATX levels and the severity of liver cirrhosis, ATX serum levels were related to the Child-Pugh stage. As illustrated in Figure 1C, patients with higher Child-Pugh stage had higher ATX levels. Moreover, there was a significant correlation between the MELD score and ATX levels (r = 0.393, P<0.001) (Figure 1D). In two patients serum samples from different time points after study inclusion were accessible. In both patients serum ATX levels stayed relatively stable. The severity of liver disease at the indicated time points is implicated by the plotted MELD scores (Figure S1).

As the MELD as well as the Child-Pugh score correlated with serum ATX levels, we examined if the serum ATX levels also correlated with individual laboratory parameters of liver function. Significant associations were found between serum ATX levels and surrogate parameters of liver synthesis capacity, namely serum albumin (r = −0.343, P<0.001) and internationalized ratio (INR) (r = 0.377, P<0.001). A significant positive correlation was also found between bilirubin, a parameter of hepatic function, and ATX (r = 0.469, P<0.001). Moreover, there were significant correlations between serum ATX and the cell death parameters, alanine aminotransferase (ALT) and aspartate aminotransferase (AST) (r = 0.324 for ALT, P<0.001; r = 0.478 for AST, P<0.001). In contrast, there were no correlations between serum levels of ATX and alkaline phosphatase (ALP) or gamma-glutamyltransferase (GGT), parameters of cholestasis (Table 2).

**Table 2:** Correlations of ATX and laboratory parameters.

Parameter	r	P value
Sodium	−0.197	0.002
Creatinine	−0.062	0.311
Thrombocytes	−0.231	<0.001
Leukocytes	−0.063	0.310
C-reactive protein	−0.019	0.760
Albumin	−0.343	<0.001
Total protein	−0.049	0.460
Bilirubin	0.469	<0.001
INR^1^	0.377	<0.001
ALT^2^	0.324	<0.001
AST^3^	0.478	<0.001
GGT^4^	−0.114	0.062
ALP^5^	0.039	0.515
LDH^6^	0.246	<0.001

Abbreviations:

1 INR, internationalized ratio;

2 ALT, alanine aminotransferase,

3 AST, aspartate aminotransferase;

4 GGT, gamma-glutaryl-transferase;

5 ALP, alkaline phosphatase;

6 LDH, lactate dehydrogenase.

### High serum ATX levels are associated with hepatic decompensation

**Figure 2:**
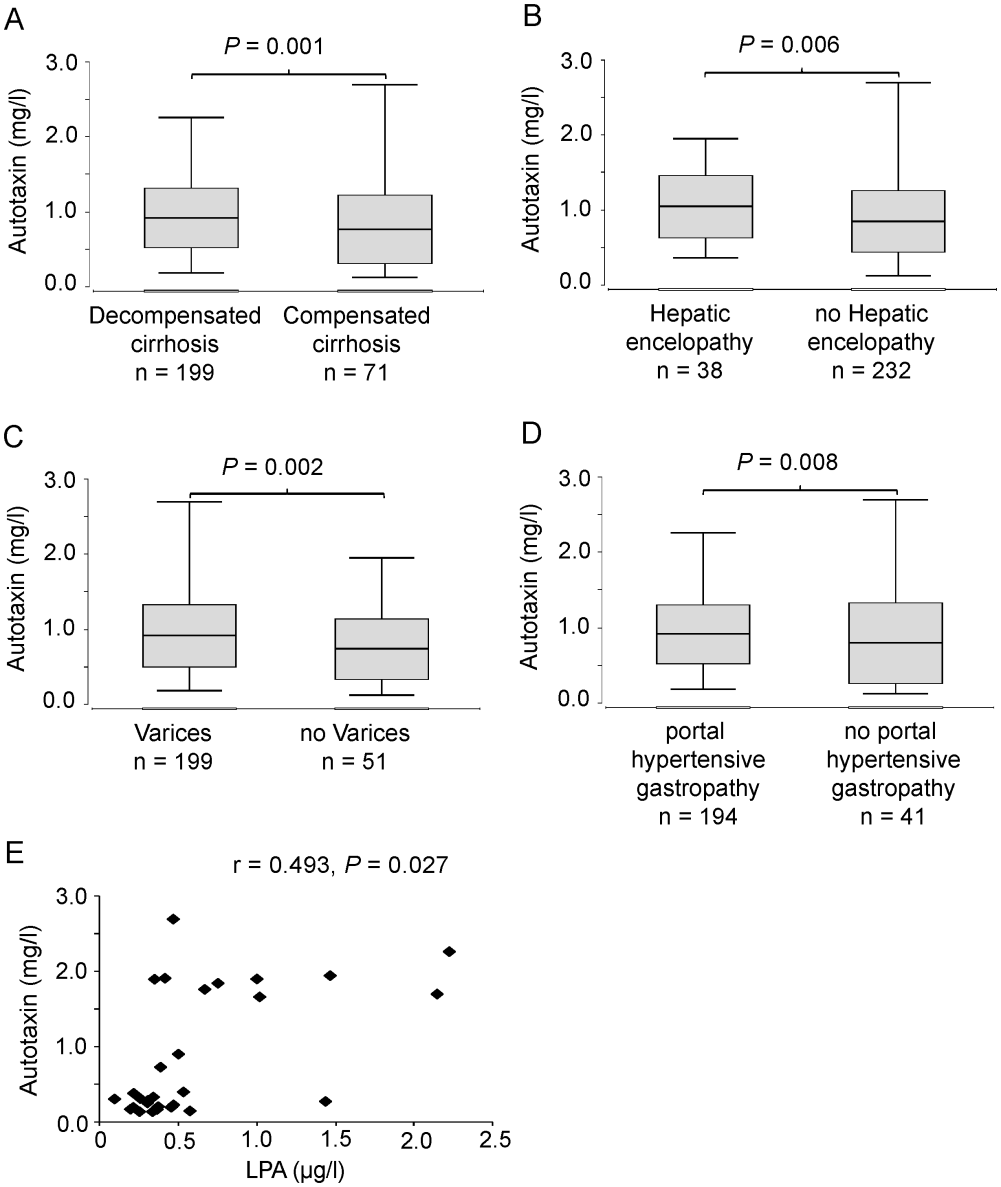
Serum autotaxin levels are higher in patients with decompensated disease and distinct complications of liver cirrhosis as compared to patients without complications. Patients with decompensated cirrhosis (A), hepatic encephalopathy (B), esophageal varices (C) and portal hypertensive gastropathy (D) show higher serum autotaxin levels. P values referring to differences between the groups were calculated with the Wilcoxon-Mann-Whitney test. (E) Correlation between LPA levels and ATX serum levels in patients with liver cirrhosis. The correlation coefficient was calculated by the Spearman test.

As ATX levels increased with advanced stages of liver cirrhosis, we investigated if ATX levels are associated with complications of liver cirrhosis. ATX levels in patients with hepatic decompensation were significantly higher than the ATX concentrations assessed in patients with compensated disease (0.825±0.396 mg/l vs. 0.669±0.456 mg/l, P = 0.001) (Figure 2A). In the subgroup analysis patients with HE showed significantly higher ATX levels than patients without this complication (0.979±0.414 mg/l vs. 0.793±0.412 mg/l, P = 0.006) (Figure 2B). ATX levels in patients that suffered from ascites, hepatorenal syndrome or spontaneous bacterial peritonitis did not differ from those without these complications (P = 0.284 for ascites, P = 0.487 for HRS, P = 0.589 for SBP).

Portal hypertension is a typical complication of liver cirrhosis. When the portal pressure increases, portosystemic shunts are arising. Major gastrointestinal shunts develop in the gastrointestinal tract presenting as gastric or esophageal varices and portal hypertensive gastropathy. As ATX is an important factor for vascular development and homeostasis the relation between ATX levels and clinical signs of portal hypertension, meaning esophageal varices and portal hypertensive gastropathy, was analyzed. Patients with esophageal varices had higher ATX levels as compared to individuals without varices (0.825±0.415 mg/l vs. 0.627±0.403 mg/l, P = 0.002) (Figure 2C). Furthermore, patients showing portal hypertensive gastropathy in esophagogastroduodenoscopy had significantly higher serum ATX levels than patients without this complication (0.829±0.393 mg/l vs. 0.629±0.533 mg/l, P = 0.008) (Figure 2D). In accordance with the fact that higher levels of ATX were found in patients with portal hypertension a negative correlation between ATX and thrombocytes were found (Table 2). A malignant complication of liver cirrhosis is HCC. As the expression of ATX in HCC appears to be an indicator for poor differentiation and vascular invasion [21]–[23], we investigated if patients with HCC show a difference in ATX serum levels. However there was no significant difference in serum ATX levels between patients with and without HCC (0.829±0.45 mg/l vs. 0.814±0.41 mg/l, P = 0.921).We also determined if the serum ATX levels correlate with its major effector LPA. Therefore, we investigated the levels of LPA in 20 sera from cirrhotic patients. As shown in Figure 2E, there was a clear correlation between the two parameters (r = 0.493, P = 0.027).

### High serum ATX levels are an independent predictor of mortality

**Figure 3:**
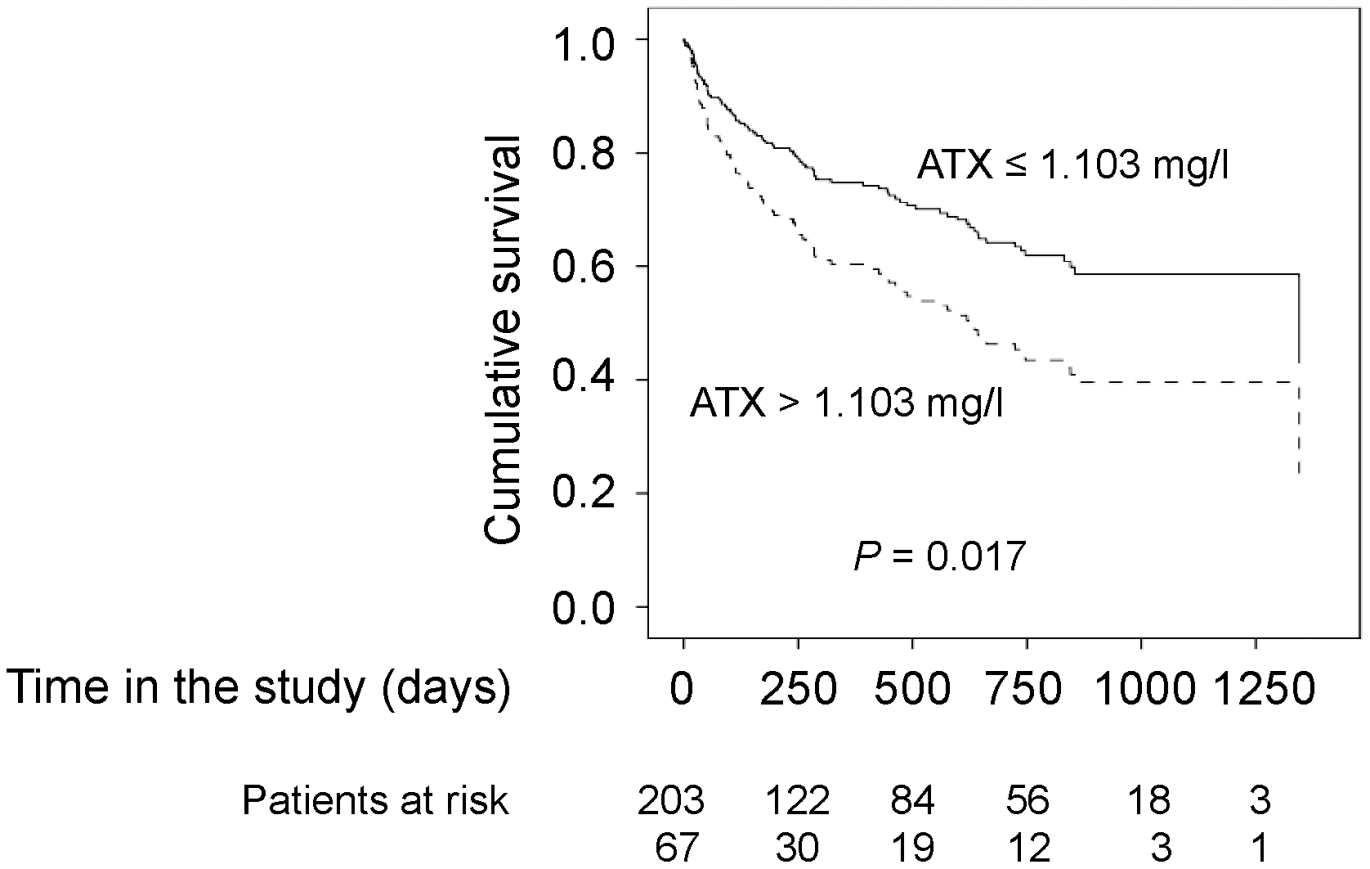
Low serum ATX levels are associated with longer survival. Survival plots show the cumulative survival of patients with high or low autotaxin serum levels. The cumulative survival was calculated with a univariate Cox regression model.

As ATX levels increased with more advanced stage of cirrhosis the prognostic impact of ATX was analyzed. The 75th percentile of serum ATX (1.103 mg/l) was defined as cut-off value and a univariate Cox regression model was performed. As shown in Figure 3 low serum ATX levels were significantly associated with longer survival (hazard ratio [HR] 0.575, 95% confidence interval [CI] 0.365–0.905, P = 0.017). In the univariate Cox regression analysis a low MELD score (≤18) as well as low levels of C-reactive protein (CRP) (<0.5 mg/dl) were also significantly associated with longer survival (Table 3).

**Table 3:** Univariate and multivariate analyses of parameters associated with overall survival.

	Univariate analysis			Multivariate analysis		
Parameter	HR	95% CI	P value	HR	95% CI	P value
Male gender	0.744	0.479–1.156	0.188			
Age ≤57 years	0.720	0.457–1.135	0.157	0.589	0.363–0.958	0.034
MELD ≤18	0.381	0.245–0.592	<0.001	0.510	0.307–0.848	0.011
Normal CRP	0.336	0.189–0.595	<0.001	0.347	0.183–0.655	<0.001
ATX ≤1.103 mg/l	0.575	0.365–0.905	0.017	0.481	0.285–0.813	0.008

Abbreviations: HR, hazard ratio; CI, confidence interval; MELD, model of end stage liver disease; Normal CRP, C-reactive protein ≤0.5 mg/dl; ATX, Autotaxin.

In order to evaluate whether serum ATX levels are an independent parameter associated with OS, we performed an age (≤57 years vs. >57 years) and gender (male vs. female) adjusted multivariate Cox regression analysis. The variables CRP (≤0.5 mg/dl vs. >0.5 mg/dl), MELD score (≤18 vs. >18) and serum ATX level (≤1.103 mg/l vs. >1.103 mg/l) were also included in the multivariate analysis. The analysis showed that lower age (*P* = 0.034), lower MELD score (P = 0.011), normal CRP (P<0.001) and low ATX levels (P = 0.008) were independently associated with OS in this cohort of cirrhotic patients (Table 3).

## Discussion

Liver cirrhosis is the result of several kinds of chronic liver damage. Our study shows an association between serum ATX levels and the stage of liver cirrhosis as well as with hepatic decompensation and specific complications of liver cirrhosis including HE, esophageal varices and portal hypertensive gastropathy. Moreover, ATX was independently associated OS. Thus, systemic ATX levels appear to indicate disease severity in cirrhotic patients.

ATX is expressed in many tissues and regulatory mechanisms that may account for an increased production of ATX have not been identified. Elevation of serum ATX levels could be due to increased production or decreased clearance of ATX from the circulation. The transcription of ATX can be stimulated by nuclear factor kappa beta (NFκB) dependent proinflammatory signaling in HCC cell lines [23]. Furthermore, elevated ATX expression has been found in HCC sections, with the highest expression in the tumor cells of HBV and especially HCV related HCC [23]. However, the source of the elevated levels of circulating ATX is still unknown. There is little data on ATX expression and its regulation in non-cancerous liver tissue. In carbon tetrachloride-induced liver fibrosis in rats ATX mRNA was not increased in the liver at eight weeks after fibrosis induction [27], which argues against an induction of hepatic ATX expression. However, it is unclear if this applies to patients. Thus, enforced expression of ATX in liver damage cannot yet be ruled out. As ATX is rapidly taken up by liver sinusoidal endothelial cells [28], reduced clearance of ATX by the damaged and fibrotic liver might explain the elevated serum ATX levels in patients with liver fibrosis [27], as well as in patients with liver cirrhosis (present study). On the other hand, serum ATX activity was also increased in hepatectomized rats [27]. An association between serum ATX and kidney function is unlikely as we did not observe a significant correlation between serum creatinine and serum ATX levels. Serum ATX levels were apparently not influenced by bacterial infections as it did not correlate with CRP levels or white blood cell count. Interestingly, the liver also plays key role in the elimination of LPA from the blood [29].

HSC are an important factor in the development of liver fibrosis and cirrhosis. On repeated or persistent liver damage they transdifferentiate into myofibroblasts [3], [4]. Progression of hepatic fibrosis is associated with an increased number of HSC [5]. LPA, which appears to be the major biological effector of ATX, inhibits apoptosis, stimulates and contracts rat HSC [8]–[10]. Therefore, one can speculate that elevated serum ATX levels are an indicator for activation of HSC during development of fibrosis and cirrhosis [15], [16]. Correlations between serum ATX activity and serum LPA levels and different stages of fibrosis were shown recently [17], [18].

The correlation between the levels of ATX and LPA in the sera was significant but unexpectedly low. This might be due to different mechanisms contributing to serum LPA levels [30]. It is also possible that the measured protein levels of ATX do not entirely reflect ATX activity levels. Further investigations will be required to clarify this issue.

Portal hypertension is a result of enhanced intrahepatic vascular resistance with activation of HSC and endothelial dysfunction as well as splanchnic vasodilatation [31]. As ATX is cleared by sinusoidal endothelial cells [28], dysfunctional endothelium may be a factor for higher blood concentrations of ATX in cirrhotic subjects. A possible causative link between the extent of portal hypertension and ATX levels is the observation that patients suffering from esophageal varices or portal hypertensive gastropathy showed significantly higher ATX serum concentrations. The gold standard for assessment of the severity of portal hypertension is the invasive hepatic venous pressure gradient (HVPG). However, only in few patients of the present cohort HVPG measurements had been performed, hampering a substantial analysis of the relation of ATX and HVPG.

Higher intrahepatic resistance and portal hypertension lead to increase in shunt blood flow. Portosystemic shunting is a major factor leading to hyperammonemia and thereby contributing to the development of HE [32]. The finding that patients suffering from HE had higher serum ATX concentrations than patients without neurological impairment stresses a potential relation between vascular dysfunction, portal hypertension and ATX blood levels.

In HCC high local ATX expression has been observed and appears to be an indicator for malignancy [21], [22]. Previously, Mazzocca et al. reported that patients with HCC have higher serum LPA levels than patients with liver cirrhosis [33]. In our study, however, the serum ATX levels did not differ significantly between patients with liver cirrhosis with or without HCC, which is in agreement with a recent study [34].

In conclusion we showed in this study for the first time that elevated serum ATX levels are associated with the stage of liver cirrhosis, the prevalence of esophageal varices, portal hypertensive gastropathy and HE. Furthermore, we showed that ATX is an independent predictor of OS. Whether the increased ATX in liver cirrhosis is caused by the disease or is a result of the reduced liver function should be further clarified.

## Supporting Information

Figure S1 Time courses of serum ATX levels and of the MELD score in two patients (A, B) with liver cirrhosis.
(TIF)
