# Supplementary figures and images for: Serum Autotaxin Is a Parameter for the Severity of Liver Cirrhosis and Overall Survival in Patients with Liver Cirrhosis – A Prospective Cohort Study
Source: PLoS One. 2014 Jul 25;9(7):e103532. doi: 10.1371/journal.pone.0103532 (PMC4111595; doi:10.1371/journal.pone.0103532)

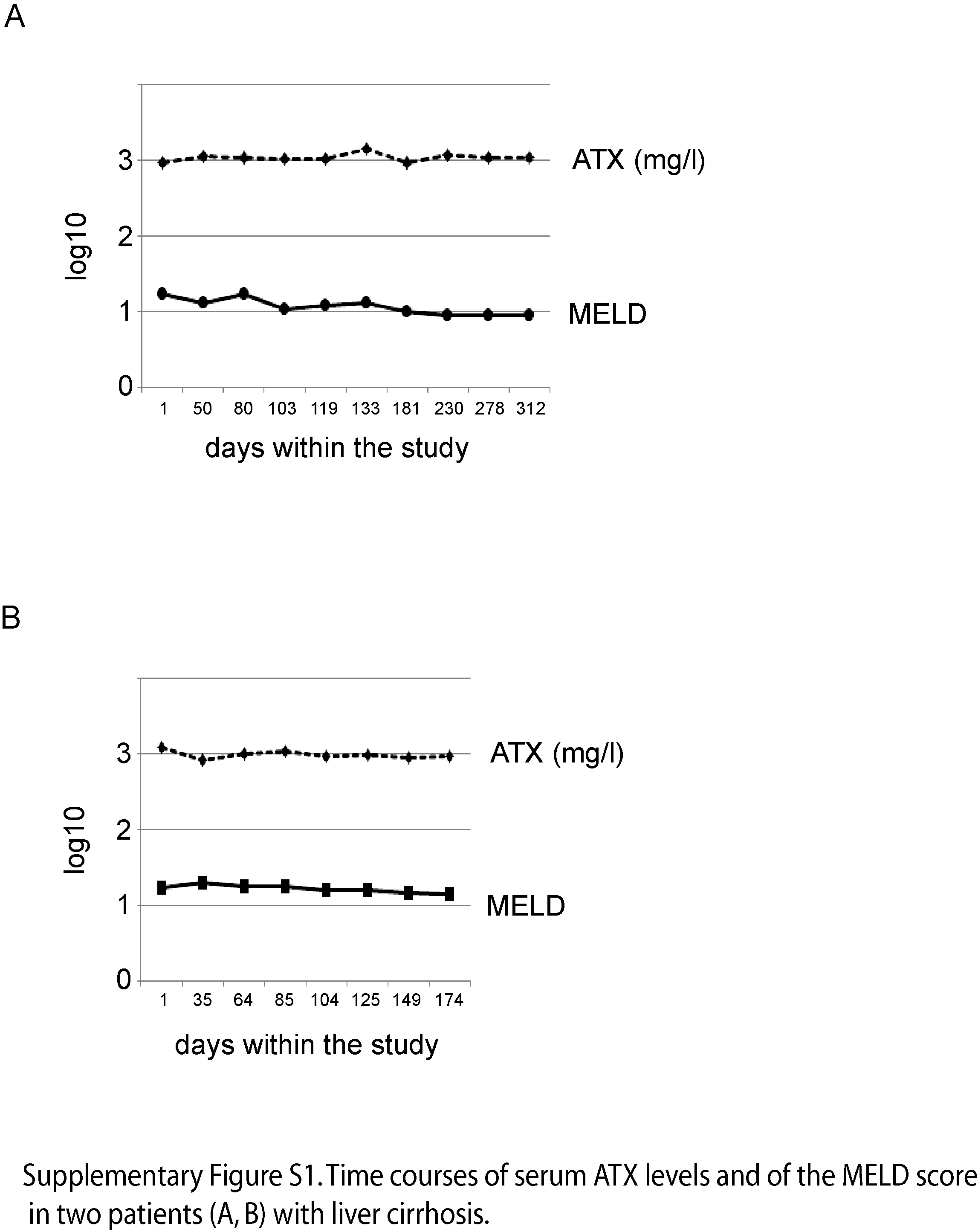

Supplement: Figure S1 — Time courses of serum ATX levels and of the MELD score in two patients (A, B) with liver cirrhosis. (TIF) [file pone.0103532.s001.tif]
